# ODNA: identification of organellar DNA by machine learning

**DOI:** 10.1093/bioinformatics/btad326

**Published:** 2023-05-17

**Authors:** Roman Martin, Minh Kien Nguyen, Nick Lowack, Dominik Heider

**Affiliations:** Department of Mathematics and Computer Science, Philipps University of Marburg, Hans-Meerwein-Str. 6, Marburg 35043, Germany; Department of Mathematics and Computer Science, Philipps University of Marburg, Hans-Meerwein-Str. 6, Marburg 35043, Germany; Department of Mathematics and Computer Science, Philipps University of Marburg, Hans-Meerwein-Str. 6, Marburg 35043, Germany; Department of Mathematics and Computer Science, Philipps University of Marburg, Hans-Meerwein-Str. 6, Marburg 35043, Germany; Center for Synthetic Microbiology (SYNMIKRO), Philipps University of Marburg, Karl-von-Frisch-Str. 14, Marburg 35043, Germany

## Abstract

**Motivation:**

Identifying organellar DNA, such as mitochondrial or plastid sequences, inside a whole genome assembly, remains challenging and requires biological background knowledge. To address this, we developed ODNA based on genome annotation and machine learning to fulfill.

**Results:**

ODNA is a software that classifies organellar DNA sequences within a genome assembly by machine learning based on a predefined genome annotation workflow. We trained our model with 829 769 DNA sequences from 405 genome assemblies and achieved high predictive performance (e.g. matthew's correlation coefficient of 0.61 for mitochondria and 0.73 for chloroplasts) on independent validation data, thus outperforming existing approaches significantly.

**Availability and implementation:**

Our software ODNA is freely accessible as a web service at https://odna.mathematik.uni-marburg.de and can also be run in a docker container. The source code can be found at https://gitlab.com/mosga/odna and the processed data at Zenodo (DOI: 10.5281/zenodo.7506483).

## 1 Introduction

A comprehensive genome of a eukaryotic species includes organellar DNA such as mitochondria, chloroplasts, or even some other plastid’s DNA. Except for some rare cases (Karnkowska *et al.* 2016), most eukaryotic cells contain at least a mitogenome. In practice, organellar DNA is often sequenced together with chromosomal genome sequencing approaches, as visualized in Supplementary Fig. S1. Based on the selected software, highly abundant organelles sequences can affect genome analyses. Therefore, methods were already developed to physically reduce putative contamination by organelles (Lutz *et al.* 2011). However, identifying organellar DNA by computational methods can also help to reduce the problem. Additionally, the preservation and identification of organellar DNA sequences can be further beneficial for performing phylogenetics or taxonomy inference analyses (Hebert *et al.* 2003, Rubinoff and Holland 2005). However, the identification remains challenging, especially if the samples are highly contaminated with bacterial sequences that can contain similar genes to those from organelles.

In most cases, there is no single rule to precisely determine if an organellar DNA sequence is within a genome assembly. Through evolution, for example, the mitogenome differentiates massively through different taxonomic clades. The mitogenome can be compact, with 11 kb in some animals, while reaching up to 1.1 Mb in some plants (Zardoya 2020). Even analyzing the gene composition is not sufficient to distinguish the correct sequences, since genes have transferred over millions of years (Kelly 2021), ending up in a set of highly conserved genes. In recent years, several tools have been developed that specifically identify organellar DNA, such as the MitoFinder (Allio *et al.* 2020) for mitochondrial or the chloroExtractor (Ankenbrand *et al.* 2018) for chloroplast DNA. These tools preferably use raw sequencing reads instead of genome assemblies, which are not always available. MitoFinder can process raw sequencing reads and genome assemblies. Recently, it was shown that some organellar DNA could be identified by MOSGA 2 (Martin *et al.* 2021) based on a combination of different multiple prediction tools.

Here, we present the software ODNA to accomplish this task and evaluate its performance. Using machine learning (ML), ODNA classifies sequences from a given eukaryotic genome assembly into nucleolar or organellar DNA. Technically, the software is a pipeline based on a pre-defined Snakemake (Köster and Rahmann 2012) workflow and MOSGA (Martin *et al.* 2020, 2021) that embeds an additional ML model for the classification.

## 2 Materials and methods

We performed 405 eukaryotic genome annotations and noted which sequences belong to organelles according to the NCBI organelles database. Based on these annotations, we completed the ML training to obtain an accurate model. The data retrieval procedure is visualized in Supplementary Fig. S2. As a result, we developed ODNA, a minimalized predefined genome annotation software based on MOSGA, which gathers the same annotation features and includes the best ML model. ODNA can classify if a sequence inside a genome assembly belongs to organellar origin.

### 2.1 Machine learning

As the feature set, ODNA annotates for each sequence in each genome assembly the repeating elements via Red (Girgis 2015), the ribosomal RNAs with barrnap, transfer RNAs with tRNAScan-SE 2 (Chan *et al.* 2021), CpG islands with newcpgreport from the EMBOSS suite (Rice *et al.* 2000), and searches against a mitochondrial and plastid gene databases from MOSGA 2 (Martin *et al.* 2021) via DIAMOND (Buchfink *et al.* 2014). Additionally, characteristics such as the GC content, sequence length, and substantial deviation from the average GC content in an assembly were encoded, as well as the density of the most features per 1 Mb. We used a stratified training-to-test ratio of 1:5 for the 10-fold cross-validation (CV) with various ML models provided by scikit-learn, including Linear Classifiers, Random Forests, k-nearest neighbors, and AdaBoost. We evaluated our model on an independent validation dataset consisting of 14 514 sequences from ten eukaryotic genome assemblies in a real-world use-case, and compared the results to Mitofinder (see Supplementary Table S1). A more detailed description is provided in the Supplementary Information. Additionally, we validated the performance of ODNA to predict chloroplast by using ten different genome assemblies with 98 882 sequences (see Supplementary Table S2). All data and scripts are available on Zenodo, ensuring reproducibility.

### 2.2 Comparison

According to our knowledge, no similar software like ODNA is freely available. Therefore, we compared the classification performance of ODNA with MitoFinder, since both software can use eukaryotic genome assemblies to identify mitochondrial sequences. For the comparison, we used a validation set of 14 514 sequences from ten eukaryotic genome assemblies that were not included in the CV (see Supplementary Table S1).

## 3 Results

In total, 405 eukaryotic genomes with 829 769 sequences were annotated for training the ML model. Among these sequences, 450 are organellar sequences. The best ML model (AdaBoost) has an MCC of 0.90 for our test data (Supplementary Fig. S3). To show exemplary our generalized model performance, ODNA can identify the mitochondrial sequence from *Cafeteria burkhardae* (Hackl *et al.* 2020), although none respective species of the taxonomical class *Bigyra* was represented neither in our training nor test data.

A comparison between MitoFinder and ODNA reveals that ODNA outperforms MitoFinder with an MCC of 0.61 versus 0.37 in identifying the mitochondrial sequence on our validation data. The higher MCC score is mainly derived from more true positives and fewer false positives, as shown in Table 1. The median execution time was shorter, with 24 min instead of 141 min. Additionally, ODNA classified the chloroplast sequences from our validation data with an MCC score of 0.73.

**Table 1.** Performance comparison between MitoFinder and ODNA in classifying mitochondrial (MT) and chloroplast (PL) DNA.^a^

Software	TP	TN	FP	FN	MCC	F1	Pre	Sen	Spe	$\tilde{t}$ (s)
MitoFinder (MT)	7	14 476	28	3	0.37	0.31	0.20	0.70	1.00	8446
ODNA (MT)	10	14 487	17	0	0.61	0.54	0.37	1.00	1.00	1447
ODNA (PL)	10	98 863	9	0	0.73	0.69	0.53	1.00	1.00	1860

a True positives (TP), true negatives (TN), false positives (FP), false negatives (FN), Matthews correlation coefficient (MCC), F1-score (F1), precision (Pre), sensitivity (Sen), specificity (Spe), and median execution time ($\tilde{t}$) are listed.

## 4 Discussion

With ODNA, we provide an easy-to-use, web-interface tool that does not require any parametrization and identifies organellar DNA precisely across all eukaryotic clades via ML. Compared to MitoFinder, ODNA is faster and more precise, demonstrating how meta-information from a DNA sequence can be used to identify meaningful biological properties. However, for the sake of completeness, we want to mention that MitoFinder’s main focus lies in identifying mitochondrial sequences and their annotation. Furthermore, since the technical pipeline is established, we consider updating our ODNA model if the NCBI genome assembly database grow.

## Supplementary Material

btad326_Supplementary_Data
